# Nyctalopia Due to Vitamin A Deficiency Secondary to Short Bowel Syndrome: When the Electroretinogram Is the Diagnostic Key

**DOI:** 10.3390/brainsci15091019

**Published:** 2025-09-20

**Authors:** Moisés León-Ruiz, Julián Benito-León, Carlos Castañeda-Cabrero

**Affiliations:** 1 Department of Neurology, Severo Ochoa University Hospital, Leganés, 28914 Madrid, Spain; 2 Department of Neurology, 12 de Octubre University Hospital, 28041 Madrid, Spain; jbenitol67@gmail.com; 3 Group of Neurodegenerative Diseases, Hospital Universitario 12 de Octubre Research Institute (i+12), 28041 Madrid, Spain; 4 Centro de Investigación Biomédica en Red Sobre Enfermedades Neurodegenerativas (CIBERNED), 28029 Madrid, Spain; 5 Department of Medicine, Faculty of Medicine, Complutense University of Madrid, 28040 Madrid, Spain; 6 Section of Clinical Neurophysiology, Department of Neurology, La Paz University Hospital, 28046 Madrid, Spain; ccastanedacabrero@yahoo.es

**Keywords:** electroretinogram, gastric adenocarcinoma, nyctalopia, photoreceptors, rods, short bowel syndrome, vitamin A deficiency

## Abstract

**Background:** Vitamin A deficiency (VAD) can occur due to malnutrition or reduced intestinal absorption, such as in short bowel syndrome (SBS). The main causes of SBS in adults include post-radiotherapy and surgery (e.g., repeated bowel resections). VAD mostly involves rods producing nyctalopia and reduced amplitudes of the electroretinogram (ERG) in scotopic conditions, with a characteristic negative ERG pattern (b/a < 1). **Case Report:** We report a 67-year-old woman with a history of gastric adenocarcinoma and several surgeries, who developed a progressive 3-month clinical picture of night blindness. **Results**: Urgent blood tests, biomicroscopy, intraocular pressure measurements, fundoscopy, and a cranial MRI were all normal. Visual evoked potentials showed increased latencies in both eyes, and full-field ERG showed a significant alteration in responses under scotopic conditions, and, to a lesser extent, under photopic conditions. Laboratory tests confirmed VAD, probably due to post-surgery and radiotherapy SBS. After parenteral vitamin replacement, VAD was clinically, analytically, and electroretinographically resolved. **Discussion:** VAD diagnosis is based on history, neuro-ophthalmological examination, and serum levels of retinol (<0.3 µg/mL) and/or retinol/retinol-binding protein (<0.8). In cases of a history of SBS, acquired nyctalopia, negative ERG, and clinical, analytical, and electroretinographic improvement with restoration of vitamin A levels, VAD should be suspected. ERG is crucial for early and appropriate management. **Conclusions:** As far as we know, this is the first reported VAD case secondary to SBS following surgical resections and radiotherapy of gastric adenocarcinoma with neuro-ophthalmological, laboratory, and electroretinographic monitoring of VAD recovery.

## 1. Introduction

Vitamin A deficiency (VAD) is rare in developed countries and may occur due to malnutrition [1] or reduced intestinal absorption [2,3], e.g., short bowel syndrome (SBS) [4]. The leading causes of SBS in adults include mesenteric ischemia, abdominal trauma, Crohn’s disease, neoplasia, post-radiotherapy enteritis, obstructive, post-surgery vascular disorders, and repeated bowel resections [4]. 11-cis-retinal (the photosensitive chromophore in photoreceptors) is derived from vitamin A, so VAD involves photoreceptors, with rods being the most vulnerable [3]. Clinically, nyctalopia (night blindness) [2] is observed, and neurophysiologically, reduced amplitudes of the electroretinogram (ERG) are noted in scotopic conditions (low absent ambient illumination and rod-driven responses), initially normal in photopic conditions (high light). An electronegative ERG pattern is characteristic (b/a < 1 [normal ≥ 2]; a-wave: photoreceptors [rods and cones] and b-wave [mainly bipolar cells, with little contribution from Müller glial cells]), due to an alteration in the post-phototransduction of the photoreceptor–bipolar cell synapse. Moreover, latencies in visual evoked potentials (VEP) may be increased [3].

We present a novel case of nyctalopia secondary to VAD, due to SBS following surgical resections and radiotherapy of gastric adenocarcinoma, with the ERG being the initial diagnostic key.

## 2. Case Report

**Figure 1:**
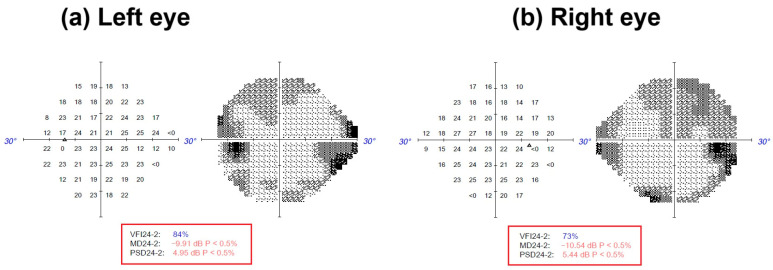
Visual field index (VFI) perimetry of the patient was obtained by standard automated perimetry (SAP) with a ZEISS Humphrey Field Analyzer 3™ (ZEISS, Jena, Germany). The 24-2 visual field test divides the central 24° into 54 threshold points. A light stimulus dims or brightens until the patient detects it, mapping retinal sensitivity across the near-periphery [5]. (**a**) Left eye: VFI24-2: 84%; MD24-2: −9.1 dB P < 0.5%; PSD24-2: 4.95 dB P < 0.5%. (**b**) Right eye: VFI24-2: 73%; MD24-2: −10.54 dB P < 0.5%; PSD24-2: 5.44 dB P < 0.5%. dB: decibel (indicates the light stimulus intensity that a patient can detect. This is a logarithmic scale, where higher dB values indicate greater sensitivity to light). MD: mean deviation. PSD: pattern standard deviation.

A 67-year-old woman with history of cataracts surgery in both eyes in 2015, bilateral Colles’ fracture in 2018, gastric adenocarcinoma with gastric surgery in 1994, and small intestine resections in 1999 and 2010 because of local tumour progression, had received chemoradiotherapy, with caused steatorrhea, treated with vitamins A, E, and B, discontinued by the patient in 2022 (due to sustained clinical improvement). She presented to the Emergency Department in October 2022 with a progressive clinical picture with 3 months of evolution, consisting of night blindness. The neuro-ophthalmological examination revealed a visual acuity of 0.63 in both eyes, with the Humphrey visual field (HVF) exam (the 24-2 test or the HVF 24-2) showing a visual field index of 84% in the left eye and 73% in the right eye (Figure 1). There were no abnormalities in urgent blood tests (including biochemistry and complete blood count), biomicroscopy, bilateral intraocular pressure (16 mmHg), fundus examination, or cranial magnetic resonance imaging (MRI) with contrast. Optical coherence tomography (OCT) revealed a mildly atrophic foveal profile (216 µm) in the right eye, with no outer retinal changes. VEP and full-field ERG were requested (for both, the normal values of the measurements and the source of those values were based on clinic norms).

Pattern-type visual evoked potentials VEP (contrast reversal) revealed bilaterally prolonged latencies without significant asymmetries (right visual pathway [averaging]: N75: 94.60 ms; P100: 143.90 ms; N145: 184.60 ms; left visual pathway [averaging]: N75: 102.30 ms; P100: 142.40 ms; N145: 184.30 ms) They were performed with a Viasys Healthcare Medelec Synergy™ (Viasys Healthcare, Conshohocken, PA, USA) by stimulating the bilateral visual pathway monocularly with a full-field contrast reversal stimulus and recording responses using surface electrodes in Oz. A sweep length of 250 ms was used, and 100 or more responses were averaged (200 in the right eye and 100 in the left eye).

Regarding VEP, the N75-wave probably represents activation of the primary visual cortex, and the subsequent P100-wave due to activation of the peristriate or the visual association cortex of the parieto-occipital brain [6]. The N145-wave could have generators at the level of the extra-striatal cortex [7].

**Figure 2:**
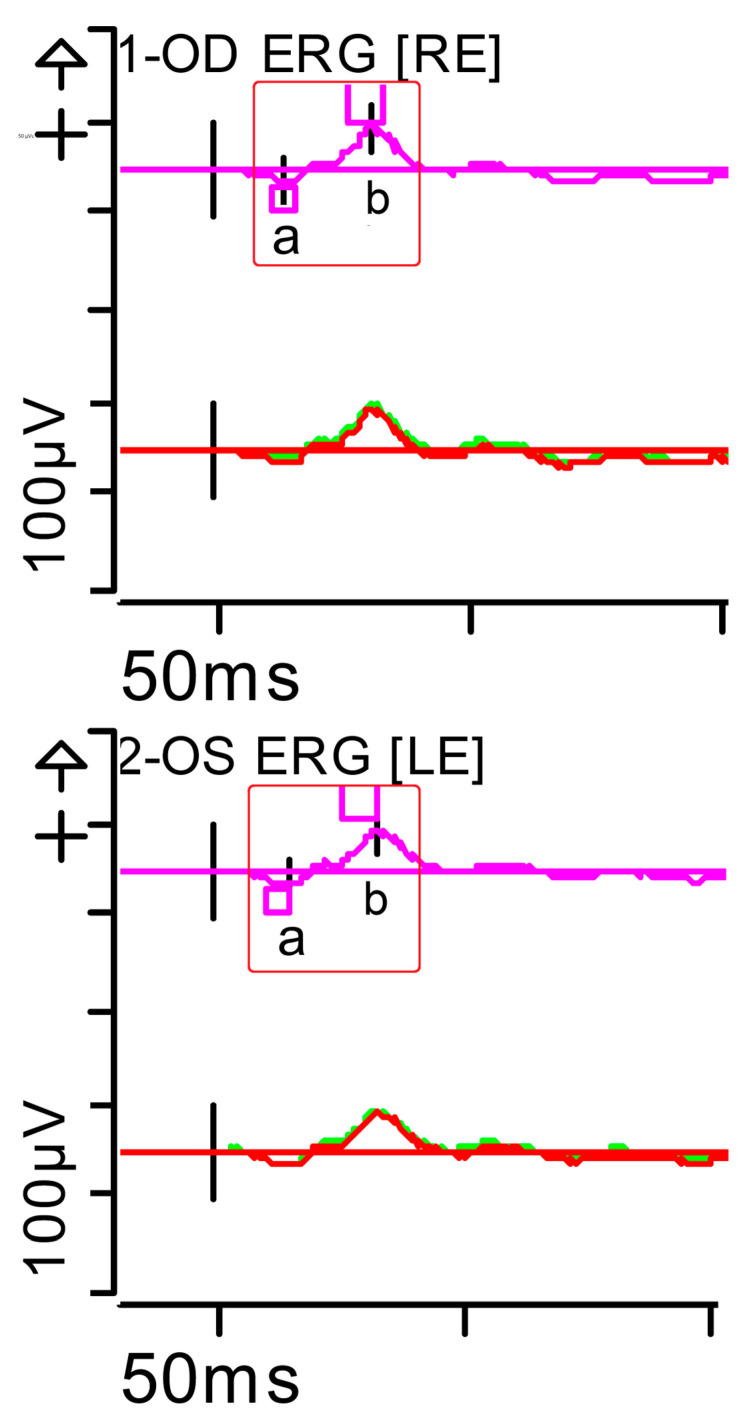
Full-field ERG at presentation. Cone response (light-adapted [LA] 3.0 candelas per square metre [cd·s·m^−2^]): peak latency (implicit time) of the a- and b-waves within normal limits, with amplitudes at the lower limits of normality (red rectangles). Step parameters: Flash. 3 (P)cd·s·m^−2^. White—6.500K. Background (Bgnd) 30 (P) cd·s·m^−2^. White—6.500K. **Markers 1-OD**: a: −12.09 µV (Norm: −29.4 ± 15.7), 15 ms (Norm: 14.1 ± 2.7); b: 59.37 µV (Norm: 118.3 ± 55.9), 32 ms (Norm: 30.5 ± 3.7). **Markers 2-OS**: a: −11.47 µV (Norm: −29.4 ± 15.7), 15.5 ms (Norm: 14.1 ± 2.7); b: 55.4 µV (Norm: 118.3 ± 55.9), 33.5 ms (Norm: 30.5 ± 3.7). Each division on the *Y*-axis represents 100 microvolts (100 μV) and each division on the *X*-axis represents 50 milliseconds (50 ms). K: Kelvin; LE: left eye; OD: oculus dexter; OS: oculus sinister; (P): photometric; RE: right eye.

**Figure 3:**
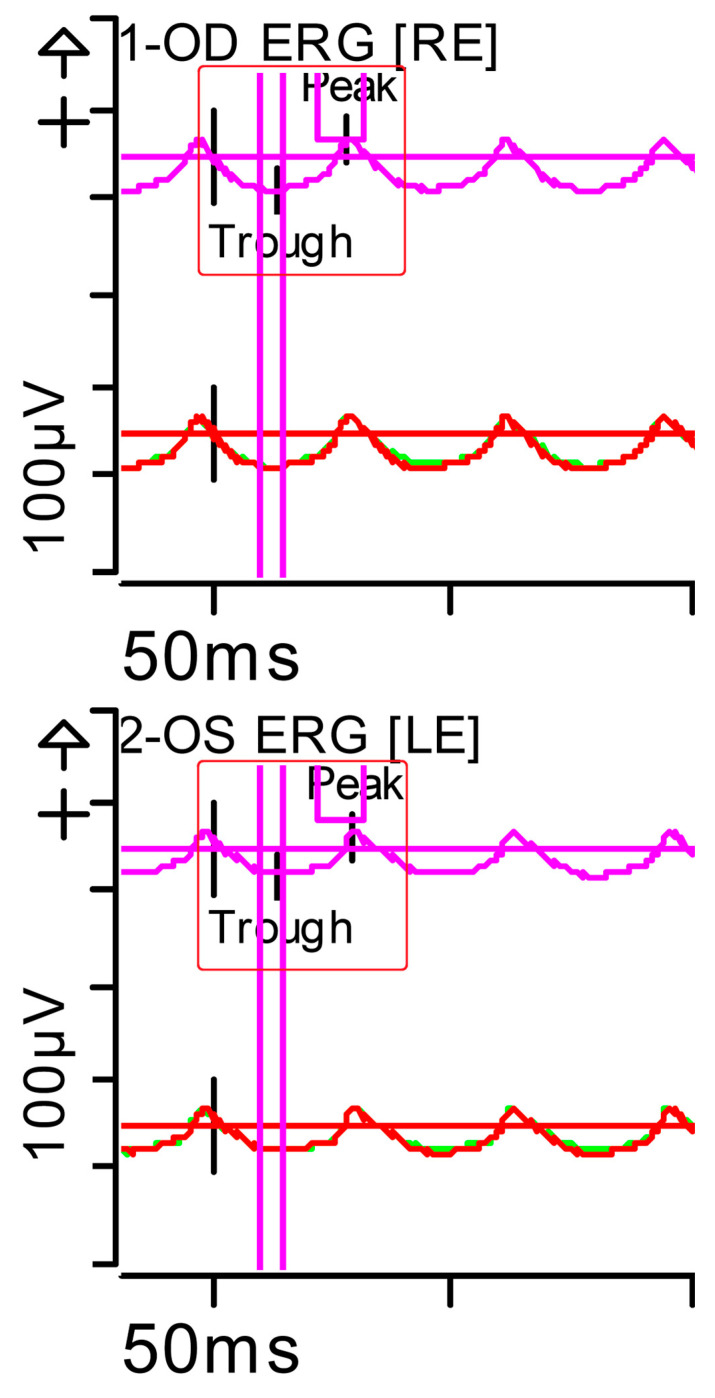
Full-field ERG at presentation. Flicker (light-adapted [LA] 30 Hz 3.0 candelas per square metre [cd·s·m^−2^]): peak and trough latencies within normal limits, with amplitudes at the lower limits of normality (red rectangles). Step parameters: Flash. 3 (P)cd·s·m^−2^. White—6.500K. Background (Bgnd) 30 (P) cd·s·m^−2^. White—6.500K. **Markers 1-OD**: Trough 13.5 ms (Norm: 12.5 ± 2.8); Peak 55.48 ms (Norm: 103.8 ± 47.4), 29 ms (Norm: 26.7 ± 4.9). **Markers 2-OS**: Trough 14.5 ms (Norm: 12.5 ± 2.8); Peak 44.79 ms (Norm: 103.8 ± 47.4), 30 ms (Norm: 26.7 ± 4.9). Each division on the *Y*-axis represents 100 microvolts (100 μV) and each division on the *X*-axis represents 50 milliseconds (50 ms). Bgnd: background; K: Kelvin; LE: left eye; OD: oculus dexter; OS: oculus sinister; (P): photometric; RE: right eye.

**Figure 4:**
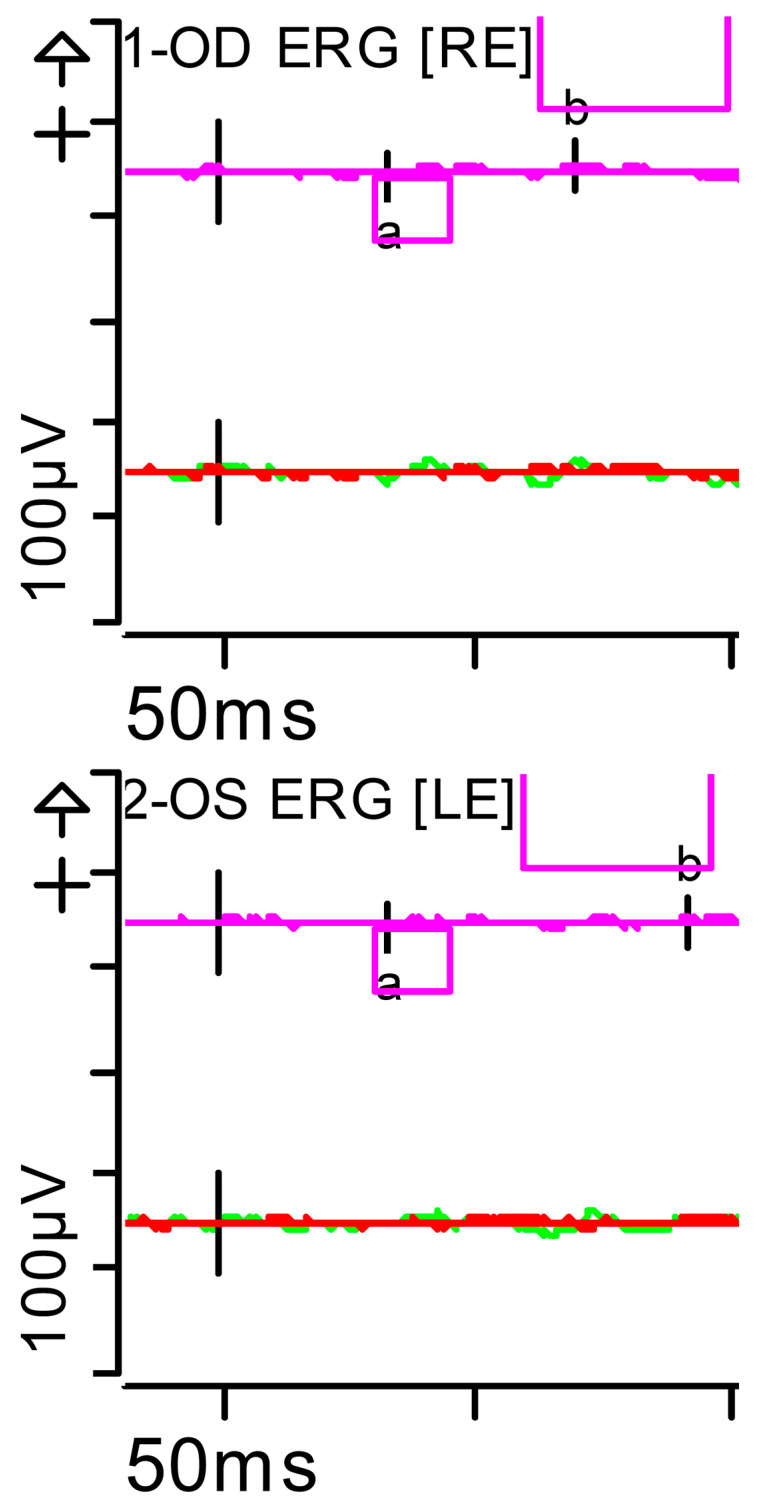
Full-field ERG at presentation. Rod response (dark-adapted [DA] 0.01 candelas per square metre [cd·s·m^−2^]): absent responses were noted. Step parameters: Flash. 0.01 (P)cd·s·m^−2^. White—6.500K. **Markers 1-OD**: a: −4.201 µV (Norm: −31.5 ± 33.9), 34 ms (Norm: 39.1 ± 8.3); b: 10.83 µV (Norm: 208.9 ± 139.3), 70.5 ms (Norm: 97.7 ± 19.5). **Markers 2-OS**: a: −2.011 µV (Norm: −31.5 ± 33.9), 33.5 ms (Norm: 39.1 ± 8.3); b: 7.877 µV (Norm: 208.9 ± 139.3), 93.5 ms (Norm: 97.7 ± 19.5). Each division on the *Y*-axis represents 100 microvolts (100 μV) and each division on the *X*-axis represents 50 milliseconds (50 ms). K: Kelvin; LE: left eye; OD: oculus dexter; OS: oculus sinister; (P): photometric; RE: right eye.

**Figure 5:**
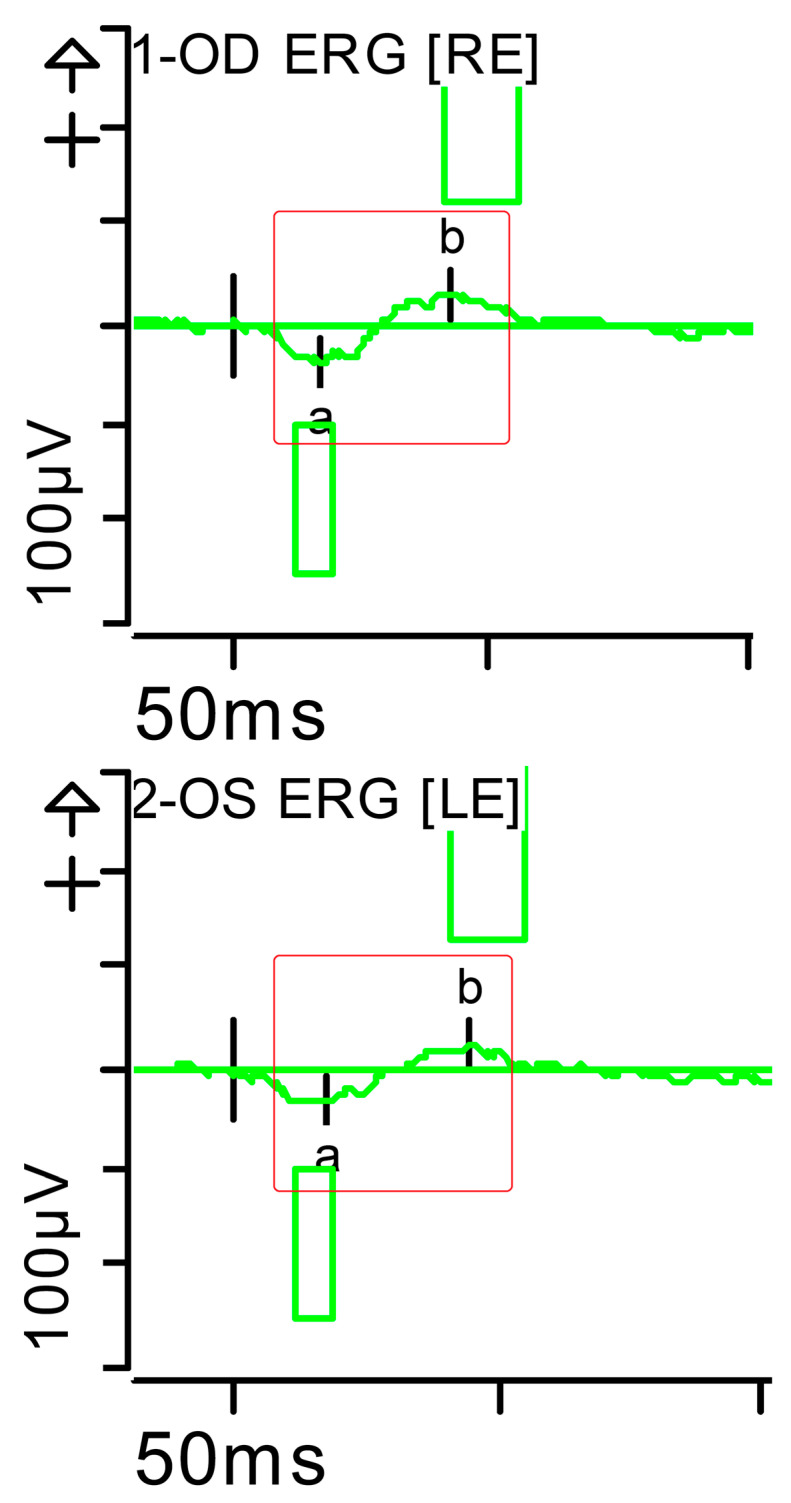
Combined response (dark-adapted [DA] 3.0 candelas per square metre [cd·s·m^−2^]): normal latencies, with severely reduced amplitudes of the a- and b-waves were noted (red rectangles). Step parameters: Flash. 3 (P)cd·s·m^−2^. White—6.500K. **Markers 1-OD**: a: −39.12 µV (Norm: −178.7 ± 80.7), 17.5 ms (Norm: 15.9 ± 4.4); b: 72.87 µV (Norm: 290.4 ± 131.2), 42.5 ms (Norm: 49.2 ± 8). **Markers 2-OS**: −34.09 µV (Norm: −178.7 ± 80.7), 18 ms (Norm: 15.9 ± 4.4); b: 60.51 µV (Norm: 290.4 ± 131.2), 46 ms (Norm: 49.2 ± 8). K: Kelvin; LE: left eye; OD: oculus dexter; OS: oculus sinister; (P): photometric; RE: right eye.

**Figure 6:**
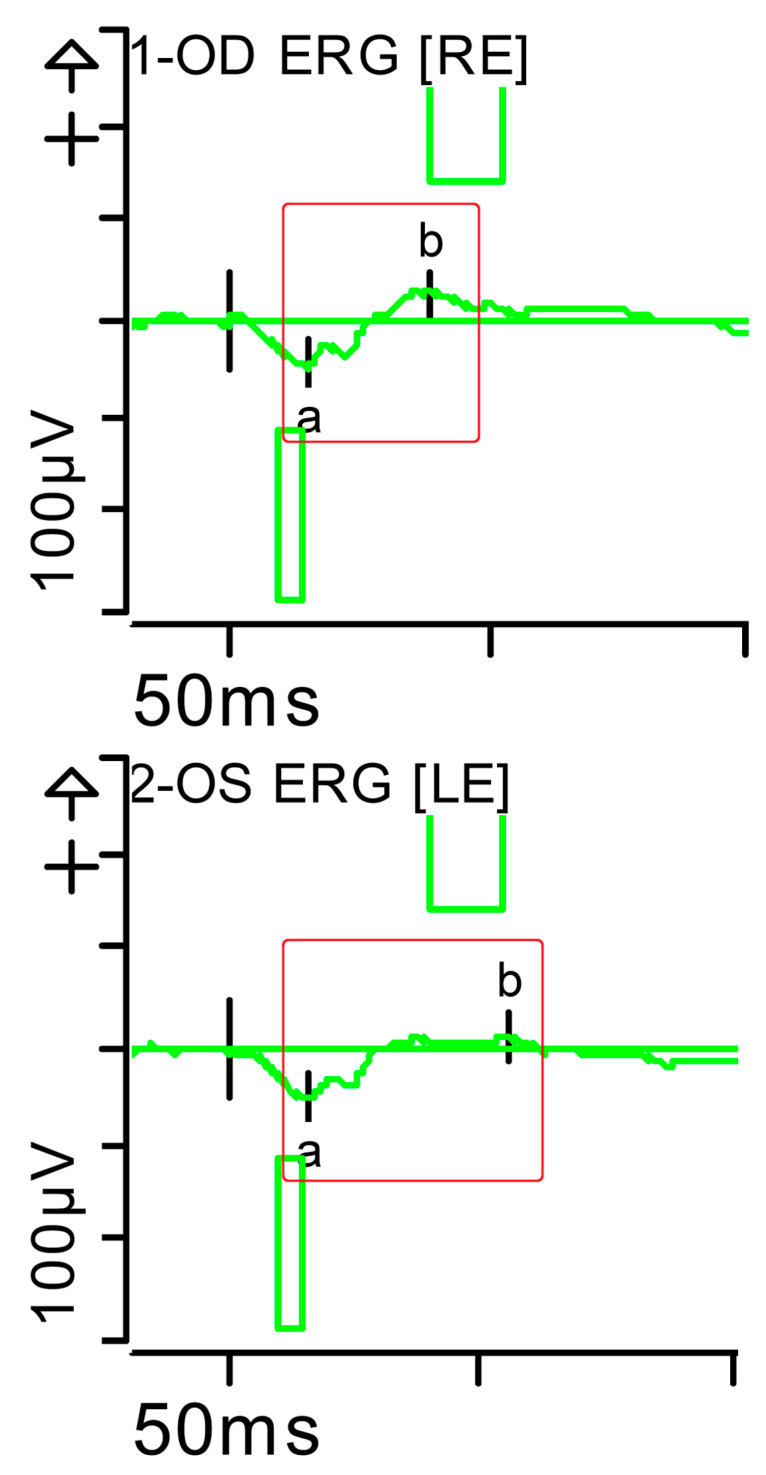
Full-field ERG at presentation. Maximum response 10 (dark-adapted [DA] 10.0 candelas per square metre [cd·s·m^−2^]): normal latencies, with severely reduced amplitudes of the a- and b-waves were noted, especially on the left side (red rectangles). Step parameters: Flash. 10 (P)cd·s·m^−2^. White—6.500K. **Markers 1-OD**: a: −47.3 µV (Norm: −200.5 ± 90.5), 16 ms (Norm: 12.5 ± 2.6); b: 76.33 µV (Norm: 297.3 ± 105.8), 40 ms (Norm: 47.2 ± 8.1). **Markers 2-OS:** a: −51.19 µV (Norm: −200.5 ± 90.5), 16.5 ms (Norm: 12.5 ± 2.6); b: 65.28 µV (Norm: 297.3 ± 105.8), 56 ms (Norm: 47.2 ± 8.1). K: Kelvin; LE: left eye; OD: oculus dexter; (P): photometric; OS: oculus sinister; RE: right eye.

**Figure 7:**
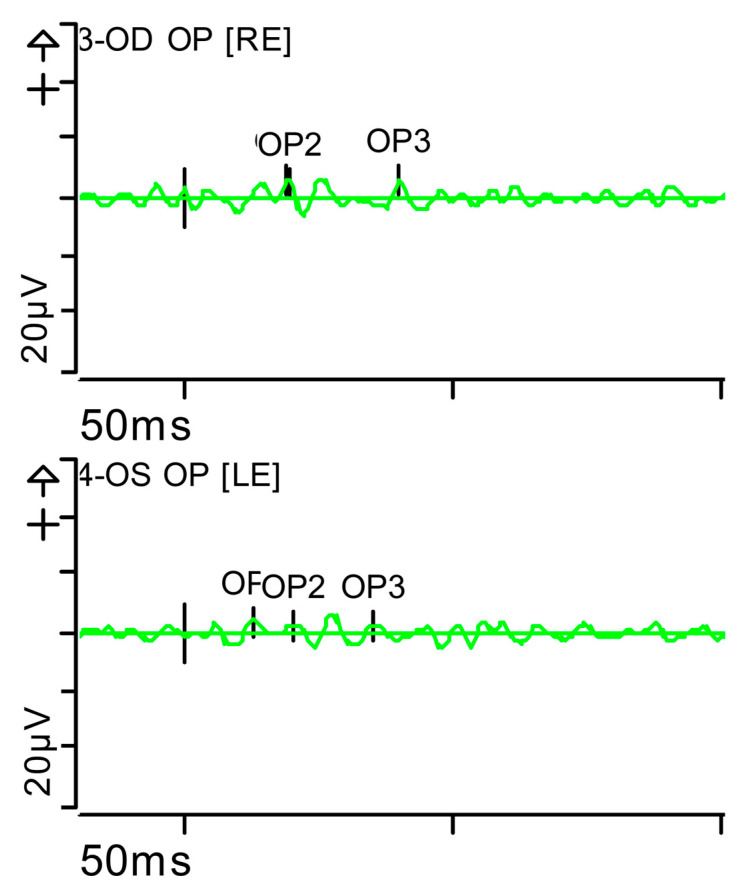
Full-field ERG at presentation. Oscillatory potentials (OP) (dark-adapted [DA] candelas per square metre 3.0 cd·s·m^−2^]): very poorly configured on both sides, hardly discernible. Oscillatory potentials: inner plexiform layer (amacrine cells). Step parameters: Flash. 3 (P)cd·s·m^−2^. White—6.500K. K: Kelvin; LE: left eye; OD: oculus dexter; OS: oculus sinister; (P): photometric; RE: right eye.

The ERG was performed in adherence with International Society for Clinical Electrophysiology of Vision (ISCEV) standards parameters (2022 update) [8], with pupil dilation and an ocular electrode (HK-LOOP type) in each eye, with a Diagnosys Red Profile™ System Espion using a ColorDome Stimulator™ and Espion™ software V6.66. Luminance dose is expressed in (P) (photopic units) candelas per second per square metre (cd·s·m^−2^). This showed a very significant alteration in responses under scotopic conditions (indicative of severe involvement of the bilateral peripheral retina [rods and ON-type bipolar cells]) and, to a lesser extent, under photopic conditions (mild involvement of the bilateral central retina [cones]). Inner retinal function, measured by oscillatory potentials generated by amacrine cells, was also reduced (Figure 2, Figure 3, Figure 4, Figure 5, Figure 6 and Figure 7 show the ERGs at presentation, i.e., before treatment). It is worth clarifying that, according to the current ISCEV (2022) standard, the b-wave response is driven primarily by the rod-driven ON-type bipolar cells [9], so the reduced rod-driven response would occur in the rod bipolar cells that are exclusively ON-type.

The patient was referred to Gastroenterology, where laboratory tests confirmed VAD (0.06 µg/mL [0.3–0.7], retinol-binding protein (RBP) (0.02 mg/dL [3,4,5,6]), vitamin A/RBP 0.5, and vitamin D deficiency (VDD) (6 ng/mL [30–100]) because of post-surgery and -radiotherapy SBS [3]. Genetic testing for hereditary causes of nyctalopia was negative.

The patient was also diagnosed with osteoporosis (T score < −2.5 SD [−2.7] by bone density scan [dual-energy X-ray absorptiometry]).

**Figure 8:**
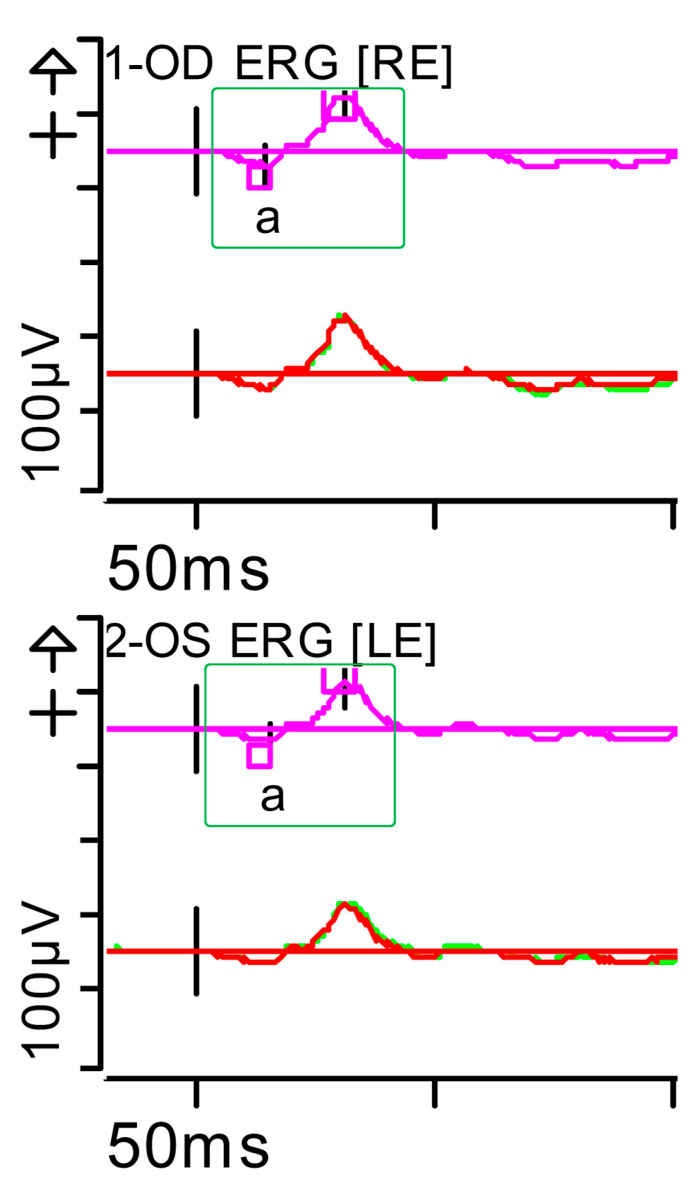
Full-field ERG at month 5. Cone response (light-adapted [LA] 3.0 candelas per square metre [cd·s·m^−2^]: peak latency (implicit time) of the a- and b-waves within normal limits, as well as their amplitudes (green rectangles). Step parameters: Flash. 3 (P)cd·s·m^−2^. White—6.500K. Background (Bgnd) 30 (P) cd·s·m^−2^. White—6.500K. **Markers 1-OD**: a: −19.98 µV (Norm: −29.4 ± 15.7), 15.5 ms (Norm: 14.1 ± 2.7); b: 93.96 µV (Norm: 118.3 ± 55.9), 31.5 ms (Norm: 30.5 ± 3.7). **Markers 2-OS**: a: −15.32 µV (Norm: −29.4 ± 15.7), 16 ms (Norm: 14.1 ± 2.7); b: 76.96 µV (Norm: 118.3 ± 55.9), 31.5 ms (Norm: 30.5 ± 3.7). Each division on the *Y*-axis represents 100 microvolts (100 μV) and each division on the *X*-axis represents 50 milliseconds (50 ms). K: Kelvin; LE: left eye; OD: oculus dexter; OS: oculus sinister; (P): photometric; RE: right eye.

**Figure 9:**
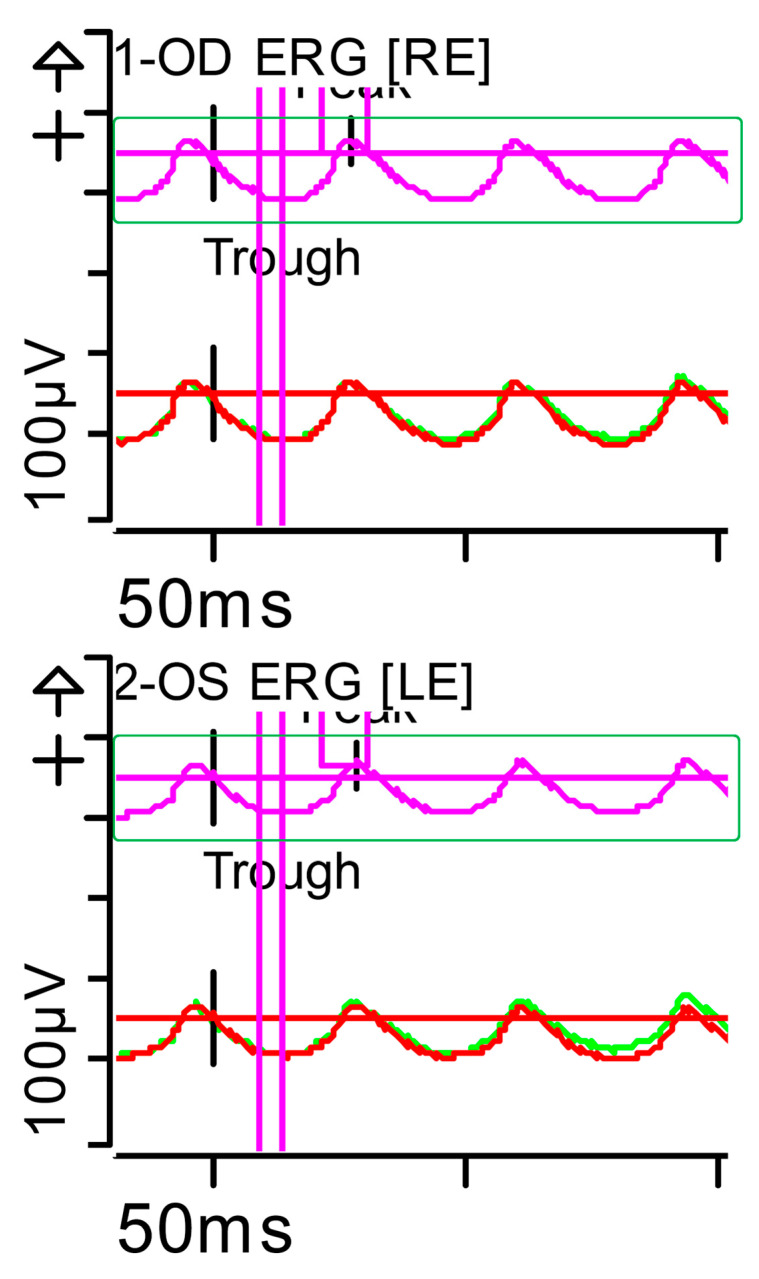
Full-field ERG at month 5. Flicker (light-adapted [LA] 30 Hz 3.0 candelas per square metre [cd·s·m^−2^]: symmetrical responses, with normal peak latency (implicit time), morphology, and peak amplitude bilaterally (green rectangles). Step parameters: Flash. 3 (P)cd·s·m^−2^. White—6.500K. Background (Bgnd) 30 (P) cd·s·m^−2^. White—6.500K. **Markers 1-OD**: Trough 14 ms (Norm: 12.5 ± 2.8); Peak 73.48 ms (Norm: 103.8 ± 47.4), 27.5 ms (Norm: 26.7 ± 4.9). **Markers 2-OS**: Trough 14.5 ms (Norm: 12.5 ± 2.8); Peak 63.34 ms (Norm: 103.8 ± 47.4), 28.5 ms (Norm: 26.7 ± 4.9). Each division on the *Y*-axis represents 100 microvolts (100 μV) and each division on the *X*-axis represents 50 milliseconds (50 ms). Bgnd: background; K: Kelvin; LE: left eye; OD: oculus dexter; OS: oculus sinister; (P): photometric; RE: right eye.

**Figure 10:**
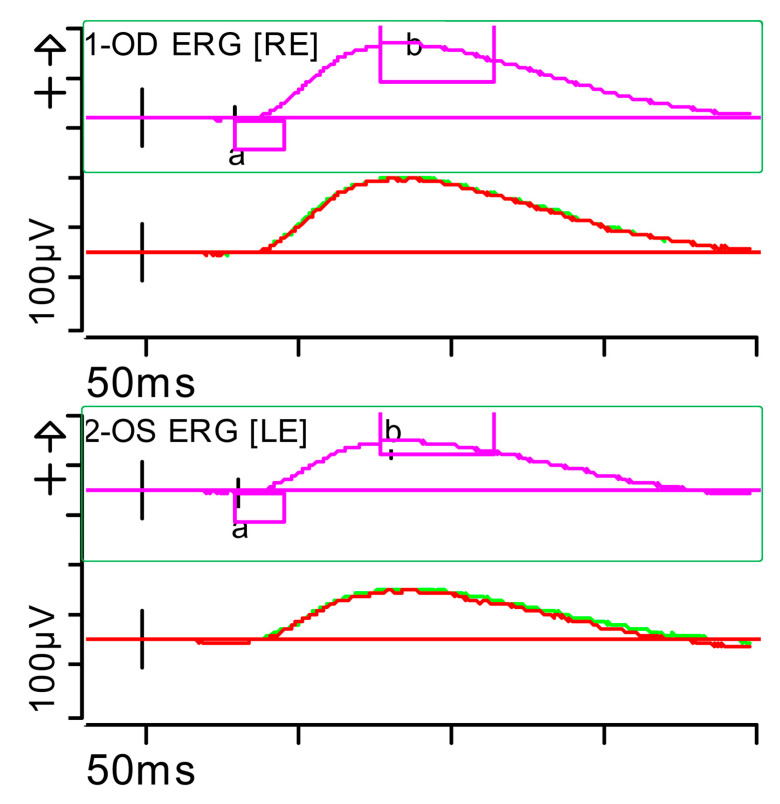
Full-field ERG at month 5. Rod response (dark-adapted [DA] 0.01 candelas per square metre [cd·s·m^−2^]): normal b-wave peak latency (implicit time) as well as their amplitudes (slight inter-side asymmetry, amplitude on the left side 32% lower than the right, not significant) (green rectangles). Step parameters: Flash. 0.01 (P)cd·s·m^−2^. White—6.500K. **Markers 1-OD**: a: −2.87 µV (Norm: −31.5 ± 33.9), 30.5 ms (Norm: 39.1 ± 8.3); b: 150.5 µV (Norm: 208.9 ± 139.3), 89 ms (Norm: 97.7 ± 19.5). **Markers 2-OS**: a: −3.661 µV (Norm: −31.5 ± 33.9), 31.5 ms (Norm: 39.1 ± 8.3); b: 102.2 µV (Norm: 208.9 ± 139.3), 81.5 ms (Norm: 97.7 ± 19.5). Each division on the *Y*-axis represents 100 microvolts (100 μV) and on the *X*-axis 50 milliseconds (50 ms). K: Kelvin; LE: left eye; OD: oculus dexter; OS: oculus sinister; (P): photometric; RE: right eye.

**Figure 11:**
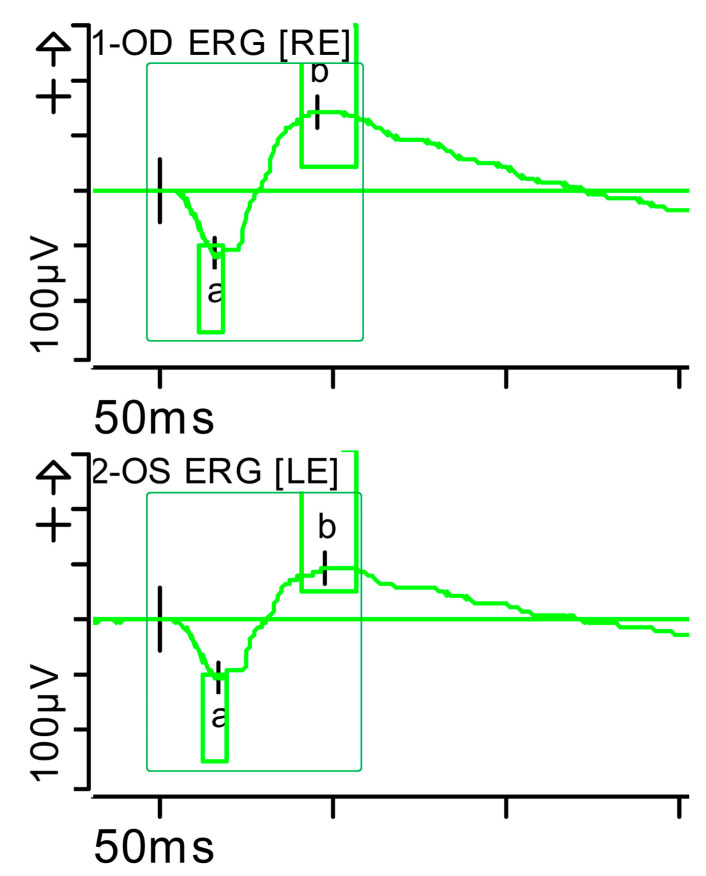
Full-field ERG at month 5. Combined response (dark-adapted [DA] 3.0 candelas per square metre [cd·s·m^−2^]): normal peak latency (implicit time) of the a- and b-waves, as well as their amplitudes (slight interocular asymmetry, amplitude on the left side 22% lower than the right, not significant) (green rectangles). Step parameters: Flash. 3 (P)cd·s·m^−2^. White—6.500K. **Markers 1-OD**: a: −117 µV (Norm: −178.7 ± 80.7), 17 ms (Norm: 15.9 ± 4.4); b: 259.7 µV (Norm: 290.4 ± 131.2), 46.5 ms (Norm: 49.2 ± 8). **Markers 2-OS**: −108.02 µV (Norm: −178.7 ± 80.7), 18 ms (Norm: 15.9 ± 4.4); b: 201 µV (Norm: 290.4 ± 131.2), 48.5 ms (Norm: 49.2 ± 8). K: Kelvin; LE: left eye; OD: oculus dexter; OS: oculus sinister; (P): photometric; RE: right eye.

**Figure 12:**
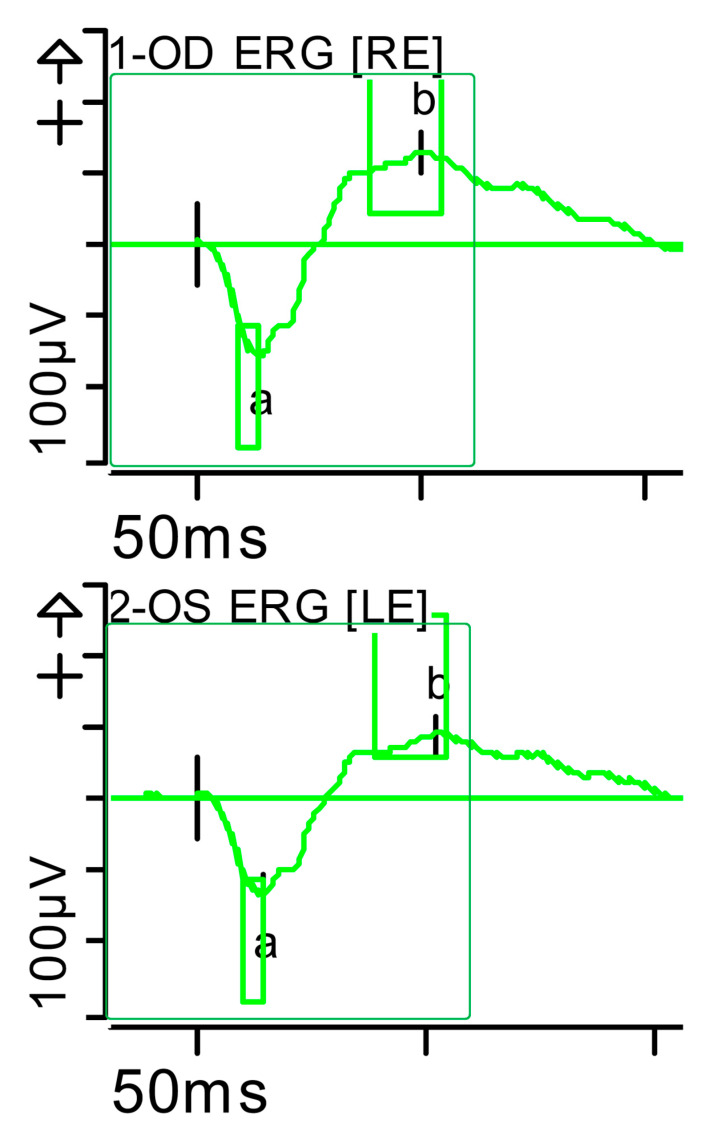
Full-field ERG at month 5. Maximum response 10 (dark-adapted [DA] 10.0 0 candelas per square metre [cd·s·m^−2^]: normal peak latency (implicit time) of the a- and b-waves, as well as their amplitudes (slight interocular asymmetry, amplitude on the left side 20% lower than the right, not significant) (green rectangles). Step parameters: Flash. 10 (P)cd·s·m^−2^. White—6.500K. **Markers 1-OD**: a: −153.4 µV (Norm: −200.5 ± 90.5), 14.5 ms (Norm: 12.5 ± 2.6); b: 281.2 µV (Norm: 297.3 ± 105.8), 51 ms (Norm: 47.2 ± 8.1). **Markers 2-OS:** a: −136.5 µV (Norm: −200.5 ± 90.5), 15 ms (Norm: 12.5 ± 2.6); b: 226.7 µV (Norm: 297.3 ± 105.8), 53 ms (Norm: 47.2 ± 8.1). K: Kelvin; LE: left eye; OD: oculus dexter; (P): photometric; OS: oculus sinister; RE: right eye.

**Figure 13:**
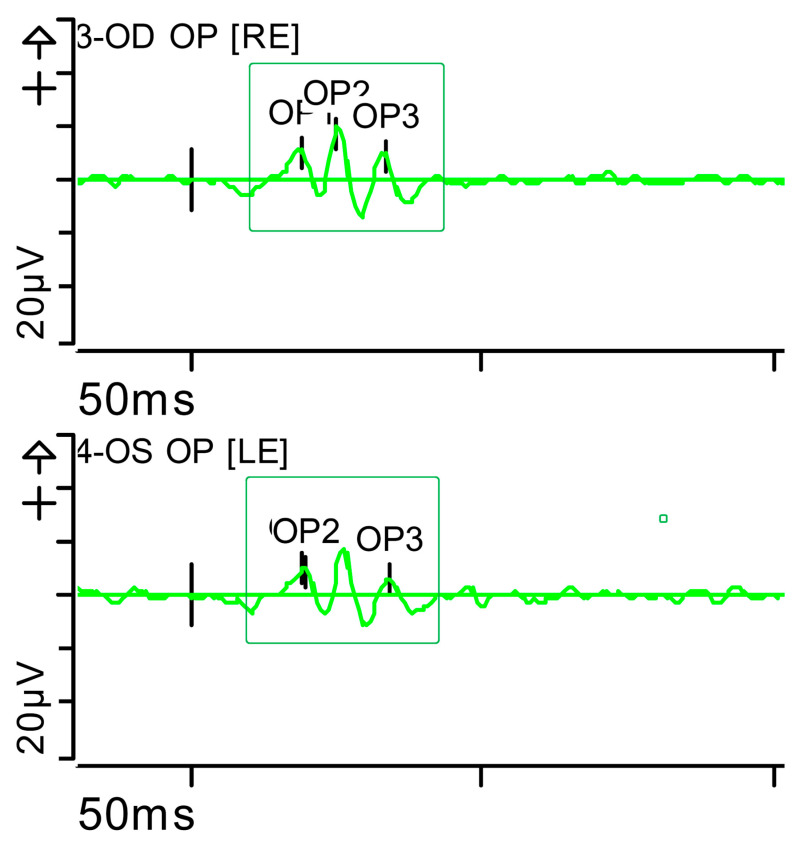
Full-field ERG at month 5. Oscillatory potentials (OP) (dark-adapted [DA] 3.0 candelas per square metre [cd·s·m^−2^] : well-configured bilaterally, with a normal number of them (green rectangles). oscillatory potentials: inner plexiform layer (amacrine cells). Step parameters: Flash. 3 (P)cd·s·m^−2^. White—6.500K. K: Kelvin; LE: left eye; OD: oculus dexter; OS: oculus sinister; (P): photometric; RE: right eye.

After parenteral (intramuscularly) multivitamin replacement (A: 100,000 IU/day 3 days; 50,000 IU/day 2 weeks, and 20,000 IU/day 8 months; D: 10,000 IU/day 8 months, later 30,000 IU/month orally), the VEP and ERG resolved at month 5 (Figure 8, Figure 9, Figure 10, Figure 11, Figure 12 and Figure 13). Notably, full-field ERG showed normalization of responses in both photopic and scotopic conditions, with no electrophysiological evidence of peripheral or central retinal involvement observed at this time. There was a slight scotopic asymmetry in the b-waves (slightly lower amplitude on the left side), but no significant difference. Compared with the previous study, a resolution of the previously described alterations was observed.

In addition, VAD resolved both clinically and analytically (also VDD analytically) at month 8, with no further symptoms related to VAD or VDD.

## 3. Discussion

Vitamin A belongs to the family of lipid-soluble compounds called retinoic acids [2]. It is crucial for cell differentiation, ocular integrity [1], and phototransduction. Its deficiency causes nyctalopia [2], but when prolonged, it can involve central and cone-mediated photopic vision [10].

VAD can cause nyctalopia, xerophthalmia, and corneal fragility and opacity [2], in addition to xerosis (dry skin), xerostomia (dry mouth), Bitot’s spots (accumulation of keratin) in the conjunctiva, white dots on the fundus (subretinal on OCT) [3], and xanthopsia (yellowish visual colour perception) [10] among other problems [2].

VAD is common in developing countries, with a prevalence of 30% in children < 5 years worldwide and almost 50% in South Asia and sub-Saharan Africa [11]. Indeed, in many developing countries, it represents a major public health issue [12].

In developed countries [2], VAD can be seen in disorders associated with fat malabsorption (cystic fibrosis, celiac disease, etc.), cholestatic liver disease (primary biliary cholangitis) [3], Crohn’s disease, SBS, bariatric surgery, etc. [13].

Deficiencies of fat-soluble vitamins, including vitamin A, are increasingly being identified in patients undergoing bariatric surgery [14]. In gastric bypass patients, VAD has been identified in 35% at 6 weeks and 18% at 1 year, with a correlation between vitamin A and prealbumin levels [15].

There is a higher risk of VAD in pregnant and breastfeeding women older than 35 years (2.74 times more likely vs. mothers aged 25–35 years), probably because they had more pregnancies and births that depleted their vitamin A stores [16].

Preformed vitamin A (retinol, retinal, retinoic acid, and retinyl esters) is hydrolyzed into retinol in the lumen of the small intestine [17], with greater absorption in the jejunum and duodenum [18].

The primary sources of vitamin A are (in order) beef liver, sweet potato, spinach, pumpkin, and carrot [19]. The activation of β-carotene to retinol in the small intestine occurs after the absorption of β-carotene from the apical membrane, and then activated retinol is transported across the basolateral membrane to the liver via chylomicrons (after esterification with palmitic acid). At the hepatic level, a trimolecular complex is formed (active retinol, RBP, and transthyretin), which prevents renal glomerular filtration [20].

The active vitamin A derivative, 11-cis-retinal, associates with the opsin protein in the retina. This complex is known as rhodopsin, the essential pigment for light perception. Through light stimulation, 11-cis-retinal is transformed into all-trans-retinal, initiating a chain of reactions whose ultimate consequence is the transmission of visual information through the optic nerve to the occipital cortex. After this reaction, part of the all-trans-retinal can be transformed back into 11-cis-retinal, allowing it to be recycled. The remaining all-trans-retinal can be transformed into retinol, which can be stored in the retinal pigment epithelium cells for reuse or converted into all-trans-retinoic acid [19].

Retinol deficiency causes nyctalopia due to deficient rhodopsin formation [21]. Cone function remains intact in the early stages, with nyctalopia being the primary visual symptom. The ERG often shows altered rod function, producing reduced dark-adapted (DA) ERG amplitudes but relatively normal light-adapted (LA) ERG amplitudes. The DA 3.0 and DA 10.0 ERGs (mixed responses) will show reduced a-wave (rods and cones), but may also show an electronegative wave (b/a < 1), reflecting the reduced response of the post-receptoral cone system [3].

The diagnosis of VAD is based on history, neuro-ophthalmological examination, and serum levels of retinol (<0.3 µg/mL) and/or retinol/RBP (<0.8) [2]. Vitamin A replacement in cases of intestinal malabsorption (as in our case) requires parenteral administration [3]. In 2011, Privet et al. reported a 55-year-old male who developed VAD after suffering from a mesenteric vein thrombosis 4 years ago, and underwent surgical 3-feet resection of his small intestine, and ascending and transverse colon [21]. In 2022, Choi et al. reported a 50-year-old female who developed VAD after bariatric surgery [20]. In both cases, VAD recovery was monitored clinically, analytically, and electroretinographically, but none was due to SBS following gastric adenocarcinoma surgery resections and radiotherapy. This case highlights that despite the cancer being in remission, side effects derived from the treatment received may appear months or even years later, involving other systems—in this case, mainly the visual system due to VAD. It also reinforces the key role of vitamin A in preserving ocular health. In our case, the increased latencies of the initial VEPs would be explained by the involvement of photoreceptors (especially rods), bipolar cells, and amacrine cells, with indirect dysfunction of ganglion cells and delayed activation of optic nerve fibres.

## 4. Conclusions

In short, in a patient with a history of SBS, acquired nyctalopia, increased VEP latencies, an electronegative ERG (with rod involvement), and clinical and electroretinographic improvement with restoration of vitamin A levels after vitamin A replacement, VAD should be suspected. The ERG is essential to facilitate rapid assessment, accurate diagnosis, and timely intervention in these cases. For this purpose, fast and effective communication between the Ophthalmology, Neurology, Clinical Neurophysiology, and Gastroenterology Departments is crucial, as shown in our case. Finally, a case–control study including patients with VAD is warranted to evaluate the sensitivity and specificity of ERG versus OCT.

## Data Availability

The data presented in this study are available on request from the corresponding author.

## Abbreviations

The following abbreviations are used in this manuscript:

DA	Dark-adapted
ERG	Electroretinogram
ISCEV	International Society for Clinical Electrophysiology of Vision
LA	Light-adapted
MRI	Magnetic resonance imaging
OCT	Optical coherence tomography
RBP	Retinol-binding protein
SBS	Short bowel syndrome
VAD	Vitamin A deficiency
VDD	Vitamin D deficiency
VEP	Visual evoked potentials
VFI	Visual field index
